# Supplementary material for: Dissecting transcriptional amplification by MYC
Source: eLife. 2020 Jul 27;9:e52483. doi: 10.7554/eLife.52483 (PMC7384857; doi:10.7554/eLife.52483)
Supplement: Supplementary file 1. [file elife-52483-supp1.docx]

**Supplementary File 1:**

gBlock1wt’

GGAGGAGAACTTCTACCAGCAGCAGCAGCAGAGCGAGCTGCAGCCCCCGGCGCCCAGCGAGGATATCTGGAAGAAATTTGAACTACTGCCCACCCCGCCCCTGTCCCCTAGTCGTCGTTCCGGGCTCTGCTCGCCCTCCTACGTTGCGGTCACACCCTTCTCCCTTCGGGGAGACAACGACGGCGGTGGCGGGAGCTTCTCCACGGCCGACCAGCTGGAGATGGTGACcgagctacTGGGtGGtGACATGGTGAACCAGAGTTTCATCTGCGACCCGGACGACGAGACCTTCATCAAAAACATCATCATCCAGGACTGTATGTGGAGCGGCTTCTCGGCcGCtGCgAAGCTCGTatctgaaAAGCTGGCCTCCTACCAGGCTGCGCGCAAAGACAGCGGCAGCCCGAACCCCGCCCGCGGCCACAGCGTCTGCTCCACCTCCAGCTTGTACCTGCAGGATCTGAGtgcagctgcatcagaatgta

gBlock1-MycBx1-AAAAA

GGAGGAGAACTTCTACCAGCAGCAGCAGCAGAGCGAGCTGCAGCCCCCGGCGCCCAGCGAGGATGCAGCAGCCGCAGCTGAACTACTGCCCACCCCGCCCCTGTCCCCTAGTCGTCGTTCCGGGCTCTGCTCGCCCTCCTACGTTGCGGTCACACCCTTCTCCCTTCGGGGAGACAACGACGGCGGTGGCGGGAGCTTCTCCACGGCCGACCAGCTGGAGATGGTGACcgagctacTGGGtGGtGACATGGTGAACCAGAGTTTCATCTGCGACCCGGACGACGAGACCTTCATCAAAAACATCATCATCCAGGACTGTATGTGGAGCGGCTTCTCGGCcGCtGCAAAGCTCGTatctgaaAAGCTGGCCTCCTACCAGGCTGCGCGCAAAGACAGCGGCAGCCCGAACCCCGCCCGCGGCCACAGCGTCTGCTCCACCTCCAGCTTGTACCTGCAGGATCTGAGtgcagctgcatcagaatgta

gBlock1-MycBx2-AAAA

GGAGGAGAACTTCTACCAGCAGCAGCAGCAGAGCGAGCTGCAGCCCCCGGCGCCCAGCGAGGATATCTGGAAGAAATTTGAACTACTGCCCACCCCGCCCCTGTCCCCTAGTCGTCGTTCCGGGCTCTGCTCGCCCTCCTACGTTGCGGTCACACCCTTCTCCCTTCGGGGAGACAACGACGGCGGTGGCGGGAGCTTCTCCACGGCCGACCAGCTGGAGATGGTGACcgagctacTGGGtGGtGACATGGTGAACCAGAGTTTCATCTGCGACCCGGACGACGAGACCTTCATCAAAAACATCATCATCCAGGCAGCTGCTGCAAGCGGCTTCTCGGCcGCtGCgAAGCTCGTatctgaaAAGCTGGCCTCCTACCAGGCTGCGCGCAAAGACAGCGGCAGCCCGAACCCCGCCCGCGGCCACAGCGTCTGCTCCACCTCCAGCTTGTACCTGCAGGATCTGAGtgcagctgcatcagaatgta

‘gBlock2wt’

AGGATCTGAGTGCAGCTGCATCAGAATGTATCGACCCCTCGGTGGTCTTCCCCTACCCTCTCAACGACAGCAGCTCGCCCAAGTCCTGCGCCTCGCAAGACTCCAGCGCCTTCTCTCCGTCCTCGGATTCTCTGCTCTCCTCGACGGAGTCCTCCCCGCAGGGCAGCCCCGAGCCCCTGGTGCTCCATGAGGAGACACCGCCCACCACCAGCAGCGACTCTGAGGAGGAACAAGAAGATGAGGAAGAAATCGATGTTGTTTCTGTGGAAAAGAGGCAGGCTCCTGGCAAAAGGTCAGAGTCTGGATCACCTTCTGCTGGAGGCCACAGCAAACCTCCTCACAGCCCACTGGTCCTCAAGAGGTGCCACGTCTCCACACATCAGCACAACTACGCAGCGCCTCCCTCCACTCGGAAGGACTATCCTGCTGCCAAGA

‘gBlock2_MbIIIx-AAAAA’

AGGATCTGAGTGCAGCTGCATCAGAATGTATCGACCCCTCGGTGGTCTTCCCCTACCCTCTCAACGACAGCAGCTCGCCCAAGTCCTGCGCCTCGCAAGACTCCAGCGCCTTCTCTCCGTCCTCGGATTCTCTGCTCTCCTCGACGGAGTCCTCCCCGCAGGGCAGCCCCGAGCCCCTGGTGCTCCATGAGGAGACACCGCCCACCACCAGCAGCGACTCTGAGGAGGAACAAGAAGATGAGGAAGCTGCAGCTGCTGCTTCTGTGGAAAAGAGGCAGGCTCCTGGCAAAAGGTCAGAGTCTGGATCACCTTCTGCTGGAGGCCACAGCAAACCTCCTCACAGCCCACTGGTCCTCAAGAGGTGCCACGTCTCCACACATCAGCACAACTACGCAGCGCCTCCCTCCACTCGGAAGGACTATCCTGCTGCCAAGA

‘gBlock2-Mb-IV-AAAA’

GATCTGAGTGCAGCTGCATCAGAATGTATCGACCCCTCGGTGGTCTTCCCCTACCCTCTCAACGACAGCAGCTCGCCCAAGTCCTGCGCCTCGCAAGACTCCAGCGCCTTCTCTCCGTCCTCGGATTCTCTGCTCTCCTCGACGGAGTCCTCCCCGCAGGGCAGCCCCGAGCCCCTGGTGCTCCATGAGGAGACACCGCCCACCACCAGCAGCGACTCTGAGGAGGAACAAGAAGATGAGGAAGAAATCGATGTTGTTTCTGTGGAAAAGAGGCAGGCTCCTGGCAAAAGGTCAGAGTCTGGATCACCTTCTGCTGGAGGCCACAGCAAACCTCCTCACAGCCCACTGGTCCTCAAGAGGTGCCACGTCTCCACACATCAGCACAACTACGCAGCGCCTCCCTCCACTCGGAAGGACTATCCTGCTGCCAAGA

gBlock3-polyA

**acctcccta**gcaaactggggcacagataat**ATCTAGATAACTGATCATAATCAGCCATACCACATTTGTAGAGGTTTTACTTGCTTTAAAAAACCTCCCACACCTCCCCCTGAACCTGAAACATAAAATGAATGCAATTGTTGTTGTTAACTTGTTTATTGCAGCTTATAATGGTTACAAATAAAGCAATAGCATCACAAATTTCACAAATAAAGCATTTTTTTCACTGCATTCTAGTTGTGGTTTGTCCAAACTCATCAATGTATCTTA**tggcctccgcgccgggttttggcgcctcccgc

gBlock4-N-terminal of c-Myc

agagctcgtttagtgaaccgtcagatcgca**CGCCACC**ATGCCCCTCAACGTTAGCTTCACTAACAGAAACTATGACCTTGACTACGACTCAGTGCAACCGTATTTCTACTGTGATGAAGAGGAAAACTTCTACCAACAGCAACAGCAA
